# Optic ataxia as a model to investigate the role of the posterior parietal cortex in visually guided action: evidence from studies of patient M.H.

**DOI:** 10.3389/fnhum.2013.00336

**Published:** 2013-07-16

**Authors:** Cristiana Cavina-Pratesi, Jason D. Connolly, A. David Milner

**Affiliations:** Department of Psychology, Durham UniversityDurham, UK

**Keywords:** optic ataxia, posterior parietal cortex, grasping, reaching, hand, arm, leg

## Abstract

Optic ataxia is a neuropsychological disorder that affects the ability to interact with objects presented in the visual modality following either unilateral or bilateral lesions of the posterior parietal cortex (PPC). Patients with optic ataxia fail to reach accurately for objects, particularly when they are presented in peripheral vision. The present review will focus on a series of experiments performed on patient M.H. Following a lesion restricted largely to the left PPC, he developed mis-reaching behavior when using his contralesional right arm for movements directed toward the contralesional (right) visual half-field. Given the clear-cut specificity of this patient's deficit, whereby reaching actions are essentially spared when executed toward his ipsilateral space or when using his left arm, M.H. provides a valuable “experiment of nature” for investigating the role of the PPC in performing different visually guided actions. In order to address this, we used kinematic measurement techniques to investigate M.H.'s reaching and grasping behavior in various tasks. Our experiments support the idea that optic ataxia is highly function-specific: it affects a specific sub-category of visually guided actions (reaching but not grasping), regardless of their specific end goal (both reaching toward an object and reaching to avoid an obstacle); and finally, is independent of the limb used to perform the action (whether the arm or the leg). Critically, these results are congruent with recent functional MRI experiments in neurologically intact subjects which suggest that the PPC is organized in a function-specific, rather than effector-specific, manner with different sub-portions of its mantle devoted to guiding actions according to their specific end-goal (reaching, grasping, or looking), rather than according to the effector used to perform them (leg, arm, hand, or eyes).

## Background

### Introduction

The defining description of optic ataxia was given by Balint (1909; see Harvey, 2005 for English translation) in his pioneering case report of a patient with extensive bilateral damage to the posterior parietal lobe (PPC). Bálint's patient had great difficulty in reaching out to take hold of objects under visual guidance: yet this was not due to a global visual difficulty, since he only reached inaccurately when he used his right arm. Presumably, therefore, the necessary visuospatial information must have been processed, to be able to guide his successful left arm. In addition, Bálint's patient could touch named parts of his own body quite accurately even with his right hand, showing that his difficulties were not simply of motor origin. Bálint accordingly concluded that the disorder must be truly visuomotor in nature, and he coined the term “optic ataxia” (*optische Ataxie*) to convey this insight. It is important to note here that Bálint's recognition of the visuomotor nature of optic ataxia (hereinafter referred as OA) was crucially dependent on the happenstance that his patient suffered from the disorder unilaterally, despite his bilateral brain damage. Had his patient suffered from reaching difficulties in both arms, like the recently much tested patient I.G. (Pisella et al., 2000) then Bálint might well have concluded that the disorder was a purely perceptual one, as Gordon Holmes did very influentially (Holmes, 1918; Holmes and Horrax, 1919), and as present-day scientists still sometimes do on the basis of patients with bilateral optic ataxia (Pisella et al., 2009).

Individual patients who present an internal behavioral dissociation (for example between left and right arms, as in this instance) provide an unrivalled opportunity to tease out the underlying nature of neuropsychological disorders, and we will argue that this is well exemplified in the patient M.H., whom we will be describing in this article. M.H. has an asymmetrical pattern of optic ataxia, such that his reaching is severely impaired only when he uses his right arm, and only when he does so to reach for targets in the right visual half-field.

During the 1980s, it became apparent that patients with optic ataxia frequently not only have difficulties with the guidance of actions within visual space, but also with accurately pre-forming their grip size when reaching to pick up objects of different sizes (Jeannerod, 1986a; Jakobson et al., 1991; Jeannerod et al., 1994). This difficulty had been prefigured by early reports that bilateral parietal-lobe lesions in monkeys caused difficulties in grasping objects. Thus, Ferrier (1886) observed that one such monkey “*always exhibited some uncertainty or want of precision in its endeavours to seize things offered it, or to pick up minute articles of food from the floor, such as currants or grains of corn*” (p. 282), and Ferrier (1890) reported in a similar monkey that “*vision gradually improved, but continued to be very imperfect, especially for minute objects, which it rarely, if ever, seized quite precisely; groping at them with the whole hand, and reaching short, or over, or to the side”* (p. 57).

In closely related work, it was reported that human optic ataxia was typically accompanied by gross errors in guiding the orientation of the wrist. This was first shown by asking patients to extricate a small object lodged in a groove that was presented at different angles from trial to trial (Tzavaras and Masure, 1976), and later by asking similar patients to pass their hand through a large oriented slot (Perenin and Vighetto, 1988). Yet control studies showed that the visual *perception* of size (Jeannerod et al., 1994), as well as of location and orientation (Perenin and Vighetto, 1988) can remain largely intact in these same patients. Thus the typical patient with optic ataxia following damage to PPC may have a broad range of visuomotor deficits, although the definition and diagnosis of optic ataxia, following Bálint, remains restricted to failures of visually guided reaching only.

An opposite pattern of visual difficulties was reported by Milner et al. (1991) in a patient suffering from a remarkably pure form of the condition known as visual form agnosia (Benson and Greenber, 1969). This patient (D.F.) has a profound difficulty in perceiving and discriminating simple shapes, or even their size or orientation, like previously-described cases of this disorder (reviewed by Heider, 2000). Yet D.F. is unimpaired in simple tasks of visuomotor control: she is indistinguishable from normal control subjects in her ability to orient her wrist when reaching to pass her hand, or post a hand-held plaque, through a slot placed at different orientations (Goodale et al., 1991; Milner et al., 1991). Similarly she is perfectly normal in tailoring her grip size during reaching movements to grasp blocks of different sizes (Goodale et al., 1991). Yet her ability to report the orientation or size of the very same target objects (whether verbally or even manually) was close to zero (Goodale et al., 1991; Milner et al., 1991). We now know from structural and functional MRI studies that DF has bilateral damage in the anterior occipital region that corresponds to the lateral occipital (LO) area in healthy subjects (James et al., 2003). This area is defined as the region that is differentially activated by viewing pictures of whole objects as opposed to fragmented versions of those same images (Malach et al., 1995; Kanwisher et al., 1997). This area within the ventral stream constitutes a pivotal hub in the perceptual analysis of objects, accounting readily for DF's visual form agnosia. Essentially her brain, lacking a functioning area LO, is no longer able to distinguish whole objects from fragmented ones.

According to the functional model of Milner and Goodale (1995, 2006), the primate brain has two somewhat distinct visual systems operating in parallel within it. One system (the so-called “ventral stream”) provides the *visual contents of our perceptual experience*, and codes information in an abstract form suitable for storage and for deploying in cognitive processes like imagining, recognizing, and planning. The other system (the so-called “dorsal stream”) serves the much more immediate function of *guiding our actions visually* from moment to moment, and therefore needs to code information in a quick, ephemeral and view-specific form. Its contents are probably not normally accessible for cognitive elaboration or conscious monitoring (Milner, 2012).

Milner and Goodale linked the two functional systems to the anatomical partition of cortical visual areas in the primate brain described by Ungerleider and Mishkin (1982). A major clue to the functional significance of this anatomically divided visual system was given in the same year by Glickstein and May (1982), who contrasted the output connections of different cortical visual areas. They reported that several dorsal visual areas send profuse downstream neuronal projections to the superior colliculus and to motor nuclei in the pons, while none of the ventral visual areas do this. These brainstem target structures in turn supply visual information to the cerebellum (the superior colliculus doing so via the pontine nuclei). Glickstein and May concluded that “*The behavioral, anatomical, and physiological evidence suggests that the parietal lobe visual areas are especially concerned with the visual guidance of movement*” (p. 136). It is these parietal visual areas, which together constitute the dorsal stream, that are damaged in patients with optic ataxia—and we believe that the systematic study of such patients can provide a valuable window into the workings of this system.

### Goal of the present review

**Figure 1:**
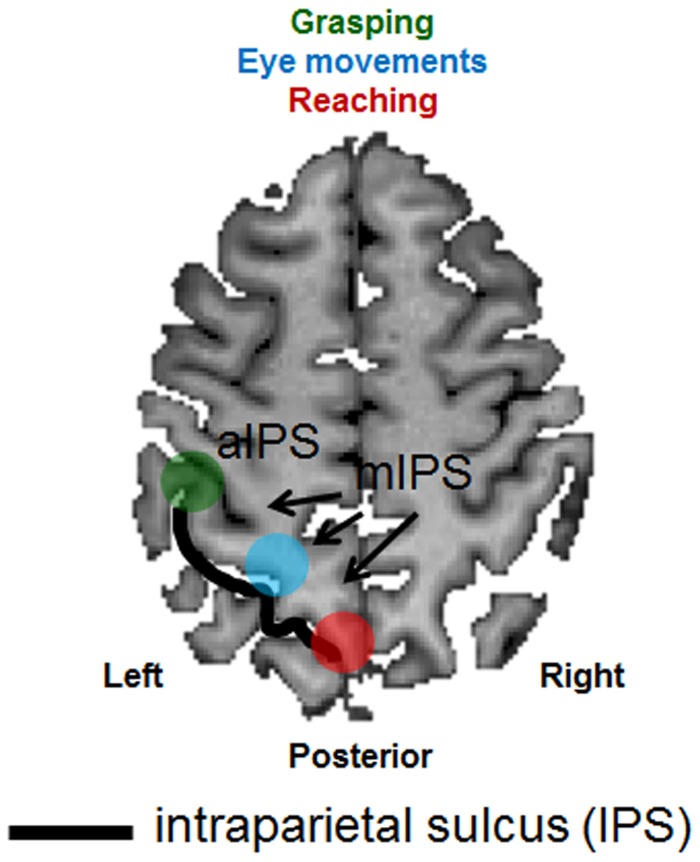
**Schematic representation of the anatomical and functional organization of the posterior parietal cortex**. An axial (horizontal) slice through the brain of a healthy individual has been chosen to depict the major sulcus within the posterior parietal cortex, the intraparietal sulcus (IPS—outlined by the use of a black line). Functional areas selective for grasping (depicted in green), reaching (depicted in red) and eye movements (depicted in light blue) have been superimposed. aIPS, anterior IPS; mIPS, medial IPS.

The overarching goal of the present review is to highlight the role played by the neuropsychological syndrome of optic ataxia in understanding the functional organization of the posterior parietal cortex (PPC). The PPC is a crucial brain structure positioned between major sensory cortices: visual cortex posteriorly and somatosensory and motor cortices anteriorly. As such, the PPC is well placed to play a critical role in the integration of sensory and motor information in the control of bodily actions. Specifically, we now know from functional neuroimaging that sub-portions of the PPC carry the visual processing necessary for the guidance of actions such as reaching, grasping and saccadic eye movements (see review by Culham et al., 2006 and section Evidence from Neuroimaging below). For example, while the more anterior portion of the intraparietal sulcus (IPS), is thought to extract visual information such as shape and size for the purpose of shaping the hands for grasping (area aIPS, highlighted in green in Figure 1), an area located posteriorly and medial to the IPS (highlighted in red in Figure 1) is thought to extract visual information for the purpose of guiding reaching through space. Finally, a portion of the PPC that is located between these two structures (highlighted in blue in Figure 1), is known to be involved in extracting the visual information necessary for guiding eye movements. Both the eye movement area and the reaching area sit medially to the IPS (mIPS). Patients with optic ataxia can provide an invaluable lesion model for understanding the nature of these systems, since different patients have different patterns of damage within the PPC, which give rise to different patterns of visuomotor deficits (see Milner and Goodale, 2006, chapter 4).

Following a lesion largely restricted to the left PPC, our patient M.H. developed a very characteristic pattern of optic ataxia. Specifically, he exhibited gross errors when reaching for a target when using his contralesional right arm, while making movements directed toward the contralesional (right) visual half-field. Given the clear-cut specificity of his deficit, whereby reaching actions are relatively spared when executed toward his ipsilateral space or when using his left arm, M.H. provides a valuable “experiment of nature” for investigating the role of the PPC. While optic ataxia is strictly defined as a disorder of reaching toward targets, its associated deficits can be studied in M.H. by examining his performance of other actions with the left and right effectors with respect to visual stimuli located in left and right halves of space. The present review will describe recent data collected in various behavioral studies using kinematic measures to record the hand, arm, and leg movements of patient M.H. (Rice et al., 2008; Cavina-Pratesi et al., 2010a; Evans et al., 2013). These results have been central to our putting forward a new hypothesis about the fundamental organizational principle within the PPC.

## Case studies with patient M.H.

### Case history

M.H. was found unconscious in the middle of the night after suffering an anoxic incident in 1995. At the time he was 42 years old and worked as a garage manager. He was diagnosed with right-side muscle weakness and raised sensory thresholds on the right side of the body. The neurological examination did not reveal any other notable problem. From anecdotal reports we know that although M.H. had no difficulty in walking or in using his arms, he did struggle in some everyday life activities. For example, he was unable to dress himself fully, particularly to fasten a necktie or shoelaces, or to put on his socks. In addition, he could not always place his mug on the table without tipping it over, and he had difficulties in walking up or downstairs without support. Despite a small improvement over the years, these problems are still present now. They have, not surprisingly, caused distress for M.H., given that as a bricklayer, a welder, and a chef he always had very good visuomotor coordination.

Later clinical assessment found evidence of right-sided extinction (Kitadono and Humphreys, 2007), mild unilateral neglect (Humphreys and Heinke, 1998; Snow et al., 2013), and impairments in spatial perception (Riddoch et al., 2004), conditions known to be often associated with PPC damage in humans. Symptoms of mis-reaching under visual guidance were first recorded during a general neuropsychological examination in which it was noted that when M.H. was asked to fixate the examiner's nose, he failed in his attempts to touch the examiner's ear. Importantly for a diagnosis of optic ataxia, he was perfectly able to point accurately to his own ear with each hand upon request [described in Kitadono and Humphreys (2007)]. Informal testing of reaching actions toward visual stimuli recorded spatial errors up to 7° when performed with the right hand toward the right side of space (Kitadono and Humphreys, 2007).

One of the first attempts to properly quantify his visuomotor behavior was performed by asking M.H. to “post” a tablet into an oriented slot (cf. Perenin and Vighetto, 1988; Milner et al., 1991). M.H. did not perform well, and even made posting errors at the mirror image orientation to the orientation of the slot. He would then use tactile contact with the slot to reorient the tablet to get the correct orientation. His actions were particularly slow when performed using the right hand (Riddoch et al., 2004). It became apparent over time that M.H. had developed an asymmetric pattern of reaching impairment in which performance was affected for stimuli presented in the right hemi-field when he was asked to use his right hand only (see Rice et al., 2008—Experiment 1). Somatosensory performance was assessed more recently (see Cavina-Pratesi et al., 2010a) using the Rivermead Assessment tests (Winward et al., 2000). M.H. scored correctly at ceiling when discriminating surface pressure on both his hands and face (used as control). His two-point discrimination on each hand was 4 mm (test 5), again within the control range. M.H. had a grating resolution threshold of 2 mm (fair, relative to a group of older controls, in Manning and Tremblay, 2006), for both hands, on a task requiring him to decide whether a grating went along or across his finger (the threshold = minimum width to make 75% discriminations). M.H. was also able to discriminate the 2.83 filament (normal) on his ipsi- and contralesional fingers on the Semmes-Weinstein monofilament test (Bell, 1984). These data are important has they indicate that there was no major somatosensory loss in either hand.

**Figure 2:**
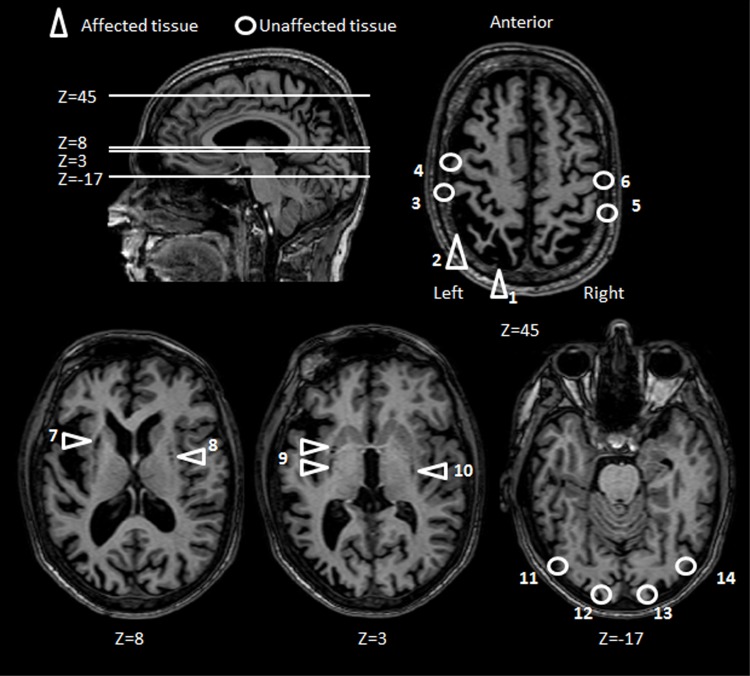
**Patient M.H.: axial brain slices**. Key areas of M.H.'s brain have been highlighted according to whether the tissue has been either affected (triangles) or left unaffected (circles) by the anoxic accident. The position of each axial slice is shown by reference to a sagittal view of M.H. brain. Areas affected by the lesion include the left PPC (1, 2), and subcortical structures (7–10). Key sensorimotor and visual areas that have been spared by the lesions include bilateral post central (3, 5), central gyri (4, 6) bilateral striate (12, 13) and extrastriate visual cortices (11, 14).

Several MRI scans performed over the years have revealed cortical atrophy of the frontal and parietal cortices (more pronounced in the left hemisphere) and subcortical atrophy bilaterally in the lentiform nucleus and in the claustrum. A large lesion is present in and surrounding the left IPS with some extension onto the medial aspects and in the inferior parietal lobule in line with current lesion overlap studies (Karnath and Perenin, 2005). It is important to emphasize that although there are adjacent regions of atrophy, right and left motor cortices appear to be intact. The occipital lobes are also unaffected (see Figure 2 for details).

In all the experiments that are described in this review, M.H. was tested at the University of Birmingham. Informed consent was obtained prior to testing. Durham University's Department of Psychology ethics advisory subcommittee approved all of the projects.

### OA affects a specific sub-category of visually guided actions (reaching but not grasping)

Early descriptions of optic ataxia provided a picture of a syndrome in which patients exhibit gross errors in reaching toward a target (particularly when located in the peripheral visual field) and in pre-shaping their hand accurately in the attempt to pick it up. While reaching movements were described as failed attempts to contact the object at its correct location, with the arm transporting the hand toward the wrong location, grasping movements in OA are typified by opening the whole hand to its maximum extent instead of pre-shaping the index finger and thumb in-flight to only the extent necessary to grasp the object. In other words the normal pre-calibration of grip size in advance of contact with an object is typically lost in patients with optic ataxia: they open their hands widely and indiscriminately, without regard for the size of the target object. As mentioned in the Introduction, grasping impairments have been associated with optic ataxia since the earliest reports of misreaching following parietal damage, in both monkeys and humans (La Motte and Acuna, 1978; Damasio and Benton, 1979; Faugier-Grimaud et al., 1985; Ferrier, 1886, 1890; Jeannerod, 1986b; Perenin and Vighetto, 1988).

Yet an obligatory co-impairment of reaching and grasping does not fit with the classic model proposed by Jeannerod 30 years ago, in which he argued that the mechanisms involved in a standard reach-to-grasp action can be partitioned into quasi-independent and separate visuomotor parts (Jeannerod, 1981). In an action such as picking up a mobile phone from a nearby table, Jeannerod proposed that the action of moving the arm to bring the hand to the object (the “transport” component), is principally influenced by visual information signaling the location of the object, whereas the concurrent anticipatory pre-shaping of the hand and fingers for grasping the phone (the “grip” component) is guided principally by the geometric properties of the object (shape and size). Although it is accepted that the two components must be somehow mutually co-ordinated, there is now extensive evidence that the transport and the grip components are each controlled online by dedicated visuomotor networks within the PPC, in association with linked systems in the premotor cortex (Jeannerod et al., 1995; Tanne-Gariepy et al., 2002; Castiello, 2005; Milner and Goodale, 2006; Castiello and Begliomini, 2008). How could we reconcile the observations of common impairments for reaching and grasping gathered in optic ataxia and the model proposed by Jeannerod (1981)?

**Figure 3:**
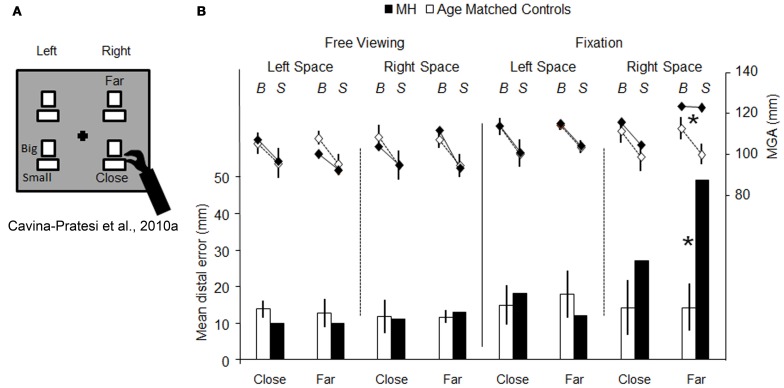
**Schematic representation of the set-up used for testing reaching vs. grasping behavior, and the results obtained**. **(A)** is a schematic representation of the set-up used by (Cavina-Pratesi et al., 2010a). The black cross depicts the fixation point and the white rectangles show the possible target locations (only one target at a time was presented) and the possible sizes of the objects to be grasped (“Big” and “Small”). Patient M.H. and age-matched controls were asked to grasp objects that could be located either close to the hand (i.e., did not require any arm reaching) or far from the hand (i.e., requiring arm reaching). Graph **(B)** summarizes the key results for both reaching (bar graph, below) and grasping (line graph, above). The bar graph depicts the reaching error as the distance between the target and the landing position (left y axis, from 0 to 50 mm) for the left and the right arm in conditions of either central fixation or free-viewing, for both M.H. (in black) and age-matched controls (in white). It should be noted that only *outward* reaching toward far objects were used in the graph (for further details please see the original article). The line graph depicts the distance between the index finger and thumb (maximum grip aperture—MGA, right y axis, from 80 to 140 mm) for grasping actions extended toward big and small objects by the left or right arm in conditions of fixation for both M.H. (in black) and age-matched controls (in white). Asterisks highlight significance differences between M.H. and controls. Errors bars depict standard deviations.

Firstly, it is entirely reasonable to argue that in most of the patients (and monkeys) studied, the lesions were extensive enough to have compromised both “grasping” and “reaching” visuomotor modules. However, the question still arises as to whether or not an impairment in grip scaling in OA necessarily implies that the patient has damage to such a “grasping” module. Instead, OA, by virtue of causing inaccurate reaching, might inevitably result in a wide anticipatory hand opening, simply to reduce the margin of error when the patient is trying to locate an object (see Wing et al., 1986 for studies of healthy participants). To cast light onto this possibility we tested patient M.H. in a paradigm that enabled us to isolate grip calibration independently of any transport component (Cavina-Pratesi et al., 2010a). By manipulating the position of the target object either far from the hand (i.e., requiring arm transport) or close to the hand (i.e., not requiring arm transport), we compared M.H.'s degree of precision in grip scaling (see Figure 3A). The rationale was that a pure grasping impairment would affect hand grasping when executed both with and without arm transport. Briefly, in this study M.H. was asked to performed grasping actions using either his right or his left arm toward objects of two different sizes (big = 5 cm or small = 3 cm) that were presented either in the right or in the left visual hemi-field. So far the paradigm does not differ from previous ones. The novelty of the design is that within each hemi-field, the target objects could be located either far away from (but still within reach), or nearby, the grasping hand (depicted as near and far in Figures 3A,B). M.H.'s performance was tested in conditions of both fixation and free viewing.

The results were clear. First, M.H. exhibited the classic pattern of OA errors during the fixation condition—gross mis-reaching when performing grasping actions using his right arm for objects presented in the right space only (see Figure 3B, data depicted using a bar graph). More importantly, these errors were present only when reaching for objects presented at the far distance. Second, and critically for present purposes, his grip calibration too was affected only when he grasped “far” objects located in right space; that is, when he executed actions with the inclusion of an arm transport (see Figure 3B, data depicted using a line graph). To be more precise, M.H. failed to calibrate his grip when reaching for objects located at the far distance within his right visual half-field, when using his right hand. In this field-hand condition he consistently opened his hand to a maximum extent without scaling for the size of the large and small objects. In contrast, the opening of his index finger and thumb (grip aperture) did faithfully reflect the size of the large and the small object for all of the three other conditions.

As in previous literature on optic ataxia, M.H.'s grip calibration failure perfectly correlates with his mis-reaching; they both appear when he reaches for far objects in his right half-field using his right hand only. Our critical finding was that M.H.'s right-handed grasping actions toward objects in the right half-field were well scaled when the action did not require any arm transport. Yet if the grip component of reaching-to-grasp movements were impaired in M.H. as a primary visuomotor deficit, then it should have manifested itself regardless of the presence or amplitude of the transport component (i.e., in both *far* and *near* conditions). That is, M.H. should have shown equally poor grip scaling with or without the inclusion of arm transport. Our evidence thus indicates that M.H.'s grasping impairment (i.e., his failure to scale his grip, and his tendency to grope for the object) is secondary to his reaching impairment. Presumably M.H., intentionally or unintentionally, compensates for the direction and distance errors resulting from his damaged visual reaching network, by habitually opening his hand widely: the wider the hand aperture, the higher the probability of successfully acquiring the object.

It could be argued that this compensatory hand opening strategy might result from the presence of degraded size information in M.H.'s peripheral visual field. Indeed “subclinical perceptual deficits in peripheral vision” have been advanced as a possible explanation for the reaching deficits observed in pure OA patients (Pisella et al., 2009). We can, however, exclude the presence of peripheral visual deficits for several reasons. First, when we tested M.H.'s ability to discriminate perceptually between the large and small objects used in the reach-to-grasp task his accuracy was very high (95% correct) and consistent across spatial locations (left, right, close, and far). Second, he did not show any deficits when asked to grasp the same objects positioned at the close location. Third, as Figure 3B demonstrates, the MGA for close objects within the impaired right hemifield did not vary between central fixation and free viewing. Finally, of course, the fact that there was no visuomotor deficit in this same retinal location when M.H. used his left hand provides conclusive internal evidence against any peripheral visual loss.

All in all, this experiment clearly confirms that extracting visual information from our environment for the purpose of visually guided actions is adversely affected in OA. More importantly however, it limits the deficit to a primary impairment in reaching. Clearly, hand shaping for the purpose of grasping the object comes into the picture as an ancillary problem in M.H. only as a secondary consequence of making inaccurate reaching movements.

### OA affects the visual control of reaching by non-targets as well as targets

**Figure 4:**
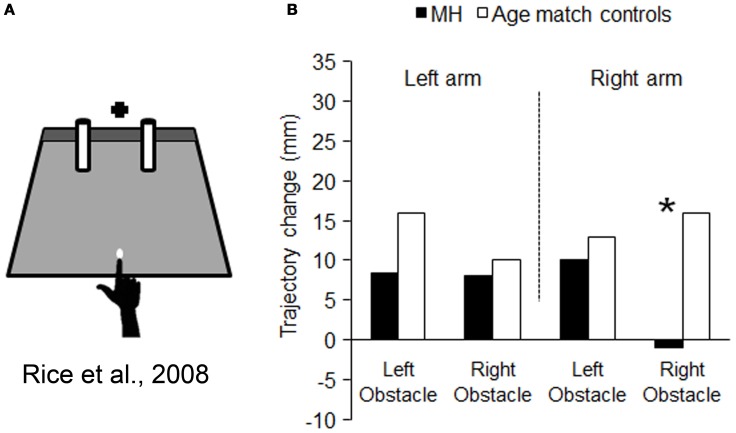
**Schematic representation of the set-up used for testing obstacle avoidance, and the results obtained. (A)** is a schematic representation of the set-up used by Rice et al. (2008). The black cross depicts the fixation point and the white dot the starting position. M.H. and controls were asked to reach to the dark gray strip at the back of the platform, passing the hand between the obstacles (cylinders). Graph **(B)** summarizes the mean amount of change in trajectory resulting from changing the location of either obstacle: in striking contrast to controls, when M.H. reached using with his right arm his trajectories were completely unaffected by the location of the obstacle within the right half-field. Asterisks highlight significance differences between M.H. and controls.

We have seen that M.H.'s optic ataxia manifests itself as a specific difficulty in calibrating reaching movements with respect to visual target locations in his peripheral visual field. The question arises, however, as to whether this difficulty is part of a more general problem in calibrating his reaches with respect to visual stimuli *in general*. For example, does M.H. have a parallel difficulty in taking into account the spatial locations of non-target visual stimuli as well as target stimuli? McIntosh et al. (2004) devised a task whereby subjects were asked to reach out between two potential obstacles, without any precisely defined target. As illustrated in Figure 4A, they merely had to touch a gray-colored strip at the back of the testing board, with no other constraint imposed other than a fixed starting point, 25 cm in front of the gray strip. The two potential obstacles consisted of two vertical rubber cylinders, each of which could appear in either of two slightly different locations. There was little danger of an actual collision with either of these cylinders, as they were always separated by at least 16 cm. Yet healthy subjects, and even patients with spatial neglect or visual form agnosia (McIntosh et al., 2004; Rice et al., 2006), have been found consistently to vary their line of their reach, slightly to the left or the right, according to the positions of these potential obstacles.

In contrast, two patients with bilateral OA failed completely to make such automatic adjustments to their reach trajectories (Schindler et al., 2004). Both of these patients, though quite different in their age and co-symptomatology, continued to make essentially identical reaches, irrespective of the changing locations of the two potential obstacles. The analysis examined separately the degree of influence of the left cylinder and right cylinder on the patients' reach trajectories—neither cylinder had any influence on the reaches. The authors concluded that our unconscious responsiveness to potential obstacles is a specific function of the PPC, specifically of the dorsal stream, which was bilaterally damaged in both patients.

If the lack of obstacle avoidance behavior in these two patients is truly a part of the same disorder as the target-guided misreaching that is characteristic of OA, then we would predict that M.H. would show a very specific pattern of results. His reaches should show a lack of influence of the *right-side cylinder only*, and show this only while using his *right arm*. As Figure 4B shows, this bizarre pattern of behavior was precisely what we observed in our study (Rice et al., 2008). When M.H. used his left arm his reaches were significantly affected by both the left and right obstacle, whereas when he used his right arm, they were affected only by the left obstacle—he remained seemingly quite oblivious of variations in the location of the right-hand object.

Given the well-defined specificity of M.H.'s optic ataxia, it seems quite compelling to infer that these two disorders of visual reaching are part and parcel of a single common disorder, namely a general inability to calibrate reaches through space using visual information of any kind. Thus, it is plausible to suggest that we have a single control system for visual reaching in the normal PPC that has at least two complementary functions: not only to guide the hand toward the locations of visual targets, but also to guide the hand *away from* the locations of visual non-targets.

### OA affects reaching actions regardless of the effector used to perform the action

In the present review, all the experiments so far have focused on testing M.H.'s abilities in performing visually guided action using his upper limbs (his arms and hands) only. This reflects to some extent the history of research in the field of optic ataxia, with an abundance of data on arm reaching along with relatively few quantitative reports on hand grasping (Jeannerod, 1986b; Jakobson et al., 1991; Jeannerod et al., 1994; Cavina-Pratesi et al., 2010a) and wrist rotation (Perenin and Vighetto, 1988). It is not clear why other effectors such as the lower limbs and the eyes have not been tested more extensively. For example, clinical evidence of deficits in visually guided lower limb movements have been described before in patients with bilateral OA, including difficulties in pointing with the toe (Rondot et al., 1977), and in descending stairs (Michel and Henaff, 2004). Understanding whether or not other effectors besides the upper limbs are affected in OA is crucial not only for a better understanding of the deficit *per se* and for its rehabilitation, but also to better understand the functional organization of the PPC. Testing the ability of OA patients to reach with their legs and compare the results with their ability to reach with their arms is crucial for testing the idea that the PPC works in an “effector specific” manner. In fact, lack of evidence of effector specificity in the PPC could lead to drastic changes in the way we understand its functional organization.

**Figure 5:**
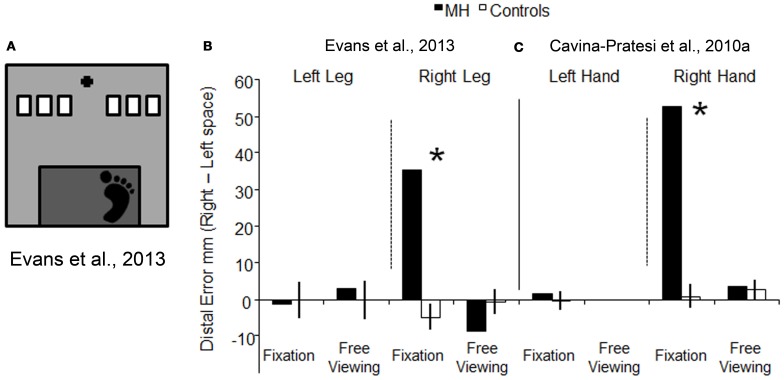
**Schematic representation of the set-up used for testing reaching with the lower limb, and a comparison between the results obtained with lower vs. upper limbs**. **(A)** is a schematic representations of the set-up used Evans et al. (2013). The black cross depicts the fixation point, the white rectangles show the possible target locations (only one target at a time was presented), and the dark gray rectangle indicates the edge of the step from which participants stepped down to make a “leg-reach.” Graph **(B)** represents the reaching error (the distance between the target and the landing position of the foot) for the left and right leg in conditions of fixation and free viewing, for both M.H. (in white) and age-matched controls (in black). Data have been plotted by subtracting the error in left space from the error in right space. **(C)** depicts the error for the upper limbs (arm) showed in graph 3b, by subtracting reaching errors made in the left visual field from errors made in the right visual field [this allows a direct comparison between the errors made with the leg in **(B)** and the arm in **(C)**]. Reaching errors executed in free viewing using the left hand are not depicted as data were not collected for that condition. For both graphs, the higher the value on the y axis, the greater the reaching error in the right half-field. Asterisks highlight significance differences between M.H. and controls.

To examine whether or not optic ataxia is effector specific, we tested patient M.H.'s ability to reach using his lower limbs. If OA is effector specific, we would predict that M.H. would exhibit a different pattern of reaching performance when using his lower limbs as compared to when he uses his upper ones. In our experiment, we asked M.H. to reach using either his left or his right leg toward targets presented either in the right or in the left visual field (Evans et al., 2013). The task was designed to resemble a natural everyday action, such as stepping down from a stair (as depicted in Figure 5A). As before, we tested M.H. in conditions of both free viewing and visual fixation. The results were clear-cut, with M.H. exhibiting gross errors in reaching toward targets in the right half-field (but not the left) only when using his right leg and in conditions of visual fixation. This pattern of results is depicted in Figure 5B, where the errors for leg reaching executed toward the left visual field have been subtracted from the errors performed when the reaching was executed toward the right visual field; the higher the value, the higher the relative error executed toward the right space. As shown in Figures 5B,C, his behavior was very similar to what we had previously found using his upper limb in Cavina-Pratesi et al. (2010a): in both studies the error was maximal when reaching were executed toward the right space using his right leg in condition of fixation only.

The pattern of results shown with the right effectors clearly suggests a similar impairment in reaching with both the upper and the lower limbs and therefore these data argue against “effector specificity.” Of course one could argue that M.H.'s extensive lesion could affect, if present, two separate modules: one dedicated to reach with the arm and the other one dedicated to reach with the leg. M.H.'s lesion encompasses most of the cortex medial to the posterior portion of the IPS and as such, it could indeed affect the functioning of portions of the cortex associated with more than one effector (see Culham et al., 2006).

In summary, this experiment suggests that M.H.'s lesion to the left medial superior parietal cortex affects reaching behavior performed with both the right upper and right lower limbs toward targets presented in the right half-field, and thus goes against the effector specific argument for the functional organization of the PPC.

## Interpreting M.H.'s results

Any conclusions reached from studies of OA have to be tempered by the fact that no two patients have an identical pattern of brain damage; therefore one can never generalize directly from one patient to another. This, however, can in some respects be a positive advantage in trying to understand how the different parts of PPC work together and separately. Given their mutual proximity (see Figure 1), the visuomotor systems within the dorsal stream in particular seem likely to be frequently co-compromised in one combination or another. It is clear that Bálint in his original description of optic ataxia, for example, was describing a patient with multiple deficits, including problems of saccadic eye movements (“psychic paralysis of gaze”) and the shifting of visual attention. However, not all patients with OA do have a problem making eye movements (e.g., Khan et al., 2009), even when their parietal damage is bilateral (e.g., Pisella et al., 2000; Michel and Henaff, 2004), nor even when they have demonstrable attentional difficulties (Michel and Henaff, 2004; Khan et al., 2009). The interesting question is what are the *obligatory* accompaniments of optic ataxia? Patient M.H.'s unusual pattern of OA provides a valuable experiment of nature that can help us to answer these questions.

The logic that we wish to use in our arguments here is based on the fact that patient M.H. presents a special case due to his unusual hand/hemi-field pattern of OA symptomatology. Although his brain damage is by no means pure, being of anoxic aetiology, his ability to reach accurately in space is remarkably well preserved in three out of four combinations: left field–left hand, left field–right hand, and right field–left hand. M.H. in other words, can act well as his own control in visuomotor studies. This allows us, for example, to argue that although he has been found to have certain attentional deficits (Kitadono and Humphreys, 2007; Snow et al., 2013) these cannot account for M.H.'s visuomotor deficits. For this, the attentional deficits would have to be shown to be highly selective; present only on the right side, but also only on the right side when M.H. is responding with his right hand. In actual fact, attentional deficits have been recorded mostly in M.H.'s *left* hemi-field (Snowden, 1992), further weakening any case that could be made. It is worth noting here also that M.H.'s attentional problems are only apparent when he is tested in a competitive attentional paradigm, such as the classical extinction paradigm. Most of the experiments described in the present review have tested M.H. with single stimuli as targets. The only exception is our study of obstacle avoidance (section OA Affects the Visual Control of Reaching by Non-targets as well as Targets above), but of course here again the pattern of deficit followed precisely the right hemifield–right hand pattern seen in M.H.'s target-directed reaching. Attentional problems could not easily have accounted for a deficit that was present when he used his right hand when reaching between potential obstacles, but that was absent when he used his left.

## Evidence from neuroimaging

Up until now, this review has focused on the neuropsychological syndrome of OA and how this has contributed to our understanding of the role played by the PPC in visually guided action. Although the OA syndrome represents a vital tool for cognitive neuroscience, neuropsychology alone obviously cannot provide a comprehensive picture. In particular, pioneering studies using electrophysiology in alert non-human primates (NHPs) in the early seventies (and up until present day), along with functional magnetic resonance imaging (fMRI) and transcranial magnetic stimulation (TMS) experiments in more recent years have provided crucially important insights. We will now highlight how our findings from patient M.H. align with recent research on visuomotor control using neurophysiology, neuroimaging, and virtual lesion techniques.

For close to four decades, the “attention *versus* intention” debate has produced a wealth of single unit recording experiments in NHPs. These provide considerable support for the argument that the PPC is organized in a highly “effector-specific fashion” for movement planning (Mountcastle et al., 1975; Shibutani et al., 1984; Seal and Commenges, 1985; Andersen, 1987; Gnadt and Andersen, 1988; Andersen, 1989; Murata et al., 1996; Shadlen, 1996; Thier and Andersen, 1996; Snyder et al., 1997; Mazzoni, 2001; Calton et al., 2002). These studies, however, overwhelmingly deal with differences between “looking” (i.e., making saccadic eye movements) and reaching with the arm. It was in the non-human primate that the candidate areas for saccade and reaching specificity in the PPC were first localized—laterally and medially to the IPS, respectively (e.g., Snyder et al., 1997; Galletti et al., 2003). Whereas the eye movement area LIP is situated midway down the lateral bank of the IPS, the reaching areas are positioned medial to this landmark. Reaching-selective neurons have been found in several foci. One such area is situated immediately posterior to the somatosensory cortices and corresponds to parietal area 5 (Buneo et al., 2002). Another area is located at the very posterior end of the PPC, area V6A (Bosco et al., 2010; Galletti et al., 2010). A third area is located midway between parietal area 5 and V6A, and has been labeled the Parietal Reach Region (or PRR, for a review see Andersen and Cui, 2009). As such, the PRR includes portions of the medial intraparietal area (or MIP) and V6A. In agreement with these studies and with relevance to OA, inactivation of the PRR induces OA-like symptomatology in NHPs and critically, does not impair saccades (Hwang et al., 2012).

It is thus not surprising that there have been many more recent attempts to examine such eye vs. hand effector-specificity non-invasively within human PPC using fMRI. While some of these studies report clear-cut differences between the eye and arm akin to the NHP data (Astafiev et al., 2003; Connolly et al., 2003; Medendorp et al., 2005; Connolly et al., 2007; Filimon et al., 2009; Hinkley et al., 2009; Van Der Werf et al., 2010; Gallivan et al., 2011) others have not (Hagler et al., 2007; Levy et al., 2007). In the latter studies, there was “overlapping” eye and hand movement activation within the PPC. However, given that certain more recent studies also failed to find a difference initially (e.g., Gallivan et al., 2011), but then using highly modern analyses tools such as multi-voxel pattern analysis (MVPA) did report a difference, such null effects must be interpreted with caution and indeed represent the overwhelming minority of the above reports. At least in the opinion of the present authors and on the basis of the above body of studies, it is reasonable to assume that there are specific regions dedicated to planning eye or arm movements in both NHPs and in the human. Nevertheless, grasping an object with the hand reliably activates an area anterior and lateral to both the eye movement and arm reaching regions of the IPS in both NHPs (Sakata et al., 1992; Murata et al., 2000; Baumann et al., 2009; Nelissen and Vanduffel, 2011; Townsend et al., 2011) and humans (Culham et al., 2003; Frey et al., 2005; Begliomini et al., 2006, 2007; Cavina-Pratesi et al., 2007; Kroliczak et al., 2007). In addition, further evidence of grasping (vs. reaching) selectivity in aIPS comes from inactivation studies in monkey (Gallese et al., 1994) and TMS studies in humans (Davare et al., 2007; Rice et al., 2007). Grasping therefore provides us with evidence for functional localization of another “effector” within the visuomotor system of the PPC, along with the arm and the eye: namely the hand itself.

Improved knowledge of the organizational (or functional) boundaries within PPC in recent years has come from studies using “around the clock” topographic memory saccade mapping paradigms (e.g., Sereno et al., 2001; Schluppeck et al., 2005). Such paradigms represent a visuomotor analogue to visual retinotopy for defining functional borders within occipital cortex (for a review, see Wandell and Winawer, 2011). These studies have revealed the existence of several representations starting from the more posterior (IPS1) to the more anterior (IPS5) portions of the PPC (Konen and Kastner, 2008; Silver and Kastner, 2009). “Around the clock” topographic saccade mapping paradigms are now considered the optimal tool with which to define early functional borders objectively within the PPC in the human. In human PPC, areas associated with programming and executing eye movements (the parietal eye fields, Connolly et al., 2000, 2005, 2007; Curtis and Connolly, 2008), programming and executing reaching movements (for a review, see Filimon, 2010), and topographic areas IPS1–IPS5 (Schluppeck et al., 2005; for review, Silver and Kastner, 2009), are all located medial to the IPS. Regardless of its specific internal subdivision, it has been suggested by others that the human superior parietal lobule (the cortex positioned medial to the IPS) may be homologous to the NHP inferior parietal lobule (Milner, 1996; Vesia and Crawford, 2012). However, it is still unclear how the functional areas associated with eye and arm movements are positioned with respect to the topographic areas IPS1–IPS5 in humans.

Although our behavioral results finding of a leg reaching impairment closely associated with an arm reaching deficit in patient M.H. are strongly suggestive, there is only one fMRI study (Heed et al., 2011a) that addresses the question of effector-specificity for leg vs. arm. This may be owing to the fact that movements of the leg have never been examined in NHPs (nor with TMS in humans for that matter). In Heed et al.'s fMRI study, “hand over eye movement” (or vice versa) activations were reported across different regions of the human PPC, akin to various previous fMRI reports already noted. However, hand and lower limb movements were also examined and these were found to be highly overlapping. This highly novel finding led the authors to argue that rather than being effector-specific (eyes, upper, and lower limbs), the PPC is instead *function* specific (saccades vs. limb transport toward a given area of space). Their results suggest that the PPC is not primarily organized for effector-specificity, but rather according to functional criteria that differ dramatically between the eyes and the limbs (arm and legs). Of course, while we physically touch the objects we reach for using the legs and the arms, the eyes never interact with their targets. Rather, saccades serve the process of “active” visual perception (Findlay and Gilchrist, 2003), whereas reaching serves grasping and manipulative actions rather than visual perception. Heed et al.'s fMRI results can certainly assist in explaining the close association between M.H.'s deficits in visually-guided leg reaching and arm reaching.

Akin to the NHP data, TMS studies provide incomplete insight into this same issue (Vesia et al., 2010; Vesia and Crawford, 2012). In the first study, the results provide support for the idea that different PPC regions have distinct roles in planning saccades and reaches in healthy humans, and this is highly consistent with NHP and fMRI data. The second study used TMS to suggest distinct neural substrates for the transport and grip formation components of reaching. Whereas the former did not test for disruption of leg movements—and is thus neither consistent nor inconsistent with our data from patient M.H., the latter study is certainly consistent with other data collected in M.H. [see section OA Affects a Specific Sub-category of Visually Guided Actions (Reaching but not Grasping)] that argues for differences in reaching vs. grasping (Cavina-Pratesi et al., 2010a). Those data add to the wealth of previous data that argue for a functional-anatomical distinction between reaching and grasping. Specifically, whereas the more anterior portions of the healthy PPC have long been associated with grasping behavior (Grafton et al., 1996) the more posterior parts of the PPC may be relatively more involved in the transport component, vis-à-vis the primate PRR/V6A. A fMRI study from Cavina-Pratesi et al. (2010b) demonstrated that while area aIPS—in the anterior portion of the PPC—is selective for grasping actions regardless of the presence of a transport component, more medial, and posterior aspects of the PPC were selective for transporting the arm. (This was true regardless of whether the subject was grasping or simply touching the object.)

## Conclusion and future directions

The primary aim of the present review was to attempt to understand how our recent OA research with patient M.H. might advance or contribute to models of how the PPC is functionally organized. Our results clearly show that patient M.H.'s impairments are restricted to the specific function of reaching, leaving his ability to grasp intact. To be specific, his visual grasping abilities remain unaffected except in circumstances that entail mis-reaching of the arm. This is a key result, insofar as for many years dual impairments of reaching and grasping have been thought to coexist within the standard definition of OA. This now appears not to be true, though of course this does not deny that a complete lesion of the dorsal stream would produce such a combined impairment. Indeed, whenever the lesion of a patient with OA includes the more anterior portion of the IPS, then we would expect a concomitant problem in grasping as well as reaching.

Another clue that brought us to think about “function” specificity in PPC was the fact that M.H.'s impairment in reaching remained regardless of the end goal of the action. For example, irrespective of whether M.H. was asked to reach for a target or to reach between a pair of potential obstacles (Rice et al., 2008), his performance was affected in a very similar manner. Just as his right hand would always land in the wrong position with respect to a target object located in right visual field, M.H. failed to take account of the locations of obstacles in his right hemispace only while using his right hand. This to say that M.H.'s inability to deal with targets was perfectly mirrored when he was asked to deal with non-targets (i.e., obstacles).

Finally, we found no evidence for effector specificity when we asked M.H. to perform reaching actions using his lower limbs as compared to his upper ones. When M.H. was asked to reach with his right leg toward targets presented in the right half-field, the magnitude of error was very similar to the same actions performed with the arm in previous experiments. While the dissociation previously found between hand grasping and arm reaching could leave open the question as to whether the PPC might work either in a “effector” or in a “function” specific manner (hand vs. arm or grasping vs. reaching), the lack of a difference in the pattern of reaching errors between the right arm (Cavina-Pratesi et al., 2010a) and the right leg (Evans et al., 2013) speaks for similar representations for reaching with both the upper and the lower limbs, and therefore for “function” specificity.

At this point we need to look outside neuropsychology and compare our results with those of other techniques. Examples of *functional* rather than *effector* specificity have come from neuroimaging studies that have shown a near perfect overlap in activations for reaching with the leg and reaching with the arm (Heed et al., 2011a), in stark contrast to activations related to eye movements. Activations for eye movements were found to be separated from both arm and leg reaching. Importantly, follow-up experiments from the same lab showed that the overlap in activation between reaching with the lower and the upper limbs is not the result of the well-known spatial resolution limits of fMRI, as more advanced adaptation designs (Heed et al., 2011b) and multivariate pattern analyses (Leone et al., 2011) could not distinguish between reaching with the arm vs. reaching with the leg in the PPC either. Interestingly, heed and co-workers (Heed et al., 2011a) found effector specificity in the frontal cortex, with activation for eye movements separated from activation for arm movements. This in turn was separated from activation for leg movements, suggesting that this region may indeed have an effector-specific organization. Collectively, however, emerging data from neuropsychology and neuroimaging support the notion that the PPC is organized in a function-specific manner, with separate portions devoted to extracting object information for guiding actions according to their specific end-goal of reaching, grasping and looking.

We can now see that although there has long been known to be “eye or hand” preference in the PPC, the leg was simply never tested. When this was tested with fMRI, the neural representations of the transport components of arm and leg reaching were found to be fully overlapping. Akin to electrophysiology, TMS studies also have yet to see whether there is “leg specificity,” or whether effective stimulation sites might simply coincide with effective arm reaching sites, as the fMRI results and M.H.'s data suggest. Based on the current evidence, although much work needs to be done, it has become apparent that “*function-*” rather than “*effector-*” specificity may be the overriding principle by which the PPC is functionally organized.

Although the present argument is supported by the converging evidence we have described, several new experiments suggest themselves for the near future. First, it will be important to test M.H. in the scanner while he performs grasping, reaching and eye movements. According to the present hypotheses we would expect normal activation in the anterior parietal cortex for grasping, but no or reduced activation for reaching (either with the arm or the leg) medially to the IPS in M.H.'s left hemisphere. Second, it will be of interest to test M.H. in two new obstacle avoidance tasks: first, one where he has to reach to *grasp* a target object in the proximity of a potential obstacle; and second, one in which he has to use his leg. In the first paradigm, when the obstacle lies just to the right during a right-arm reach by a healthy subject, there is a reliable reduction in the maximum grip aperture of the hand as it approaches the target (Mon-Williams et al., 2001; Rice et al., 2006, Experiment 1). It seems likely that this effect will depend critically on the integrity of the “grasp area” aIPS. Thus, we would predict that although M.H. shows his characteristic selective deficit when tested for obstacle avoidance in the McIntosh *reaching* paradigm (see section OA Affects the Visual Control of Reaching by Non-targets as well as Targets above), he should show uniformly intact avoidance behavior in this *grasp* paradigm. Such a dissociation would offer powerful support for our interpretations. Our second proposal will test M.H.'s ability to avoid obstacles using his legs. Our prediction must be that in a paradigm analogous to that developed by McIntosh et al. (2004), M.H. should show an impairment only when using his right foot, and only with respect to potential obstacles located on his right side. Such a finding would further support our hypothesis of function specificity.

Third, we hope to test additional patients with asymmetrical patterns of OA to examine the generalizability of M.H.'s results. Of course patients with the specialized pattern of unilateral OA that M.H. has are very rare and difficult to spot in the standard clinical setting. Indeed it is well known that OA patients in general do not overtly complain about their inability to reach using vision, presumably because they unwittingly compensate by gazing at the target before acting upon it.

However, other patients with unilateral parietal damage would be predicted to show the same degree of tight coupling between arm reaching, leg reaching, and obstacle avoidance as we have seen in M.H. For example, we predict that a patient who shows a “field effect” only (cf. Perenin and Vighetto, 1988) when performing reaches with either arm, will show the same field effect (only) in leg reaching and obstacle avoidance. Of course such a patient considered alone would not be as compelling as M.H., as their pattern of OA is much more common. Nonetheless the prediction needs to be tested on such patients if our conclusions are to be vindicated.

It will also be potentially interesting to test such new patients using the above-mentioned task of grasping in the presence of an obstacle, according to whether or not the patient has a true primary grasping deficit (e.g., due to concomitant damage to area aIPS) or not. This can be determined using the paradigm described in section OA Affects a Specific Sub-category of Visually Guided Actions (Reaching but not Grasping) above. We would predict that those patients who do have such a primary grasp deficit should show an obstacle avoidance impairment in both the reach and grasp paradigms, whereas those like M.H. who do not show a primary grasp deficit should show a deficit only in the reaching paradigm.

Of course we do not in this review wish to claim that these regions in the human dorsal stream are *purely* visuomotor in function. Indeed we know from numerous previous studies that somatosensory functions are well represented and indeed may share some degree of common sensorimotor mapping with the visual modality (e.g., Azanon et al., 2010). The relevance of these representations to optic ataxia *per se*, however, is currently unclear. For example, the fact that M.H. can re-orient a tablet to pass it through a slot presented at different orientations by relying on tactile contact (Riddoch et al., 2004) shows that he can use somatosensory cues to perform tasks that he cannot perform under visual guidance. A recent study has reported misreaching within the proprioceptive domain in two OA patients (Blangero et al., 2007), though of course this does not imply that proprioceptive loss is an integral component of optic ataxia *per se*, even in those two patients. Certainly it is difficult to see how such a loss could result in the kind of highly selective reaching disorder we see in M.H., whereby errors are confined to the right hand operating in right visual hemispace.

In conclusion, we believe that there is much of theoretical importance to be gained by testing patients with asymmetrical patterns of optic ataxia. They allow a much more analytic approach to be taken to questions of visuomotor control than can be taken with bilateral cases. But aside from the theoretical interest of our findings with M.H., we believe that they also speak to issues of practical relevance. For example, our technique for teasing apart deficits in reaching and grasping should be useful for researchers to use with future patients in visuomotor studies. Secondly, if we are right that the problem in descending stairs that patients with OA often complain of is due to “optic ataxia of the leg(s)” (section OA Affects Reaching Actions Regardless of the Effector Used to Perform the Action above), then clinicians could usefully take this into account when planning appropriate rehabilitation programmes.

### Conflict of interest statement

The authors declare that the research was conducted in the absence of any commercial or financial relationships that could be construed as a potential conflict of interest.
